# (*E*)-Methyl *N*′-[1-(4-methoxy­phen­yl)ethyl­idene]hydrazinecarboxyl­ate

**DOI:** 10.1107/S1600536808024264

**Published:** 2008-08-06

**Authors:** Lu-Ping Lv, Wei-Ping Yu, Wen-Bo Yu, Xue-Feng Zhou, Xian-Chao Hu

**Affiliations:** a Department of Chemical Engineering, Hangzhou Vocational and Technical College, Hangzhou 310018, People’s Republic of China; b Zhejiang Xinan Chemical Industrial Group Co. Ltd, Jiande 311604, People’s Republic of China; c Research Center of Analysis and Measurement, Zhejiang University of Technology, Hangzhou 310014, People’s Republic of China

## Abstract

The mol­ecule of the title compound, C_11_H_14_N_2_O_3_, adopts a *trans* configuration with respect to the C=N bond. The dihedral angle between the benzene ring and the hydrazinecarboxyl­ate plane is 12.06 (9)°. Mol­ecules are linked into a one-dimensional network by N—H⋯O hydrogen bonds and C—H⋯π inter­actions. The benzene rings of inversion-related mol­ecules are stacked with their centroids separated by 3.777 (1) Å, indicating π–π inter­actions.

## Related literature

For general background, see: Parashar *et al.* (1988 ▶); Hadjoudis *et al.* (1987 ▶); Borg *et al.* (1999 ▶). For related structures, see: Shang *et al.* (2007 ▶).
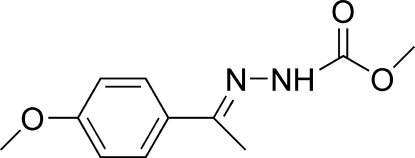


## Experimental

### 

#### Crystal data

C_11_H_14_N_2_O_3_
*M* *_r_* = 222.24
Monoclinic, 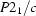

*a* = 12.416 (3) Å
*b* = 11.113 (3) Å
*c* = 8.073 (2) Å
β = 95.628 (3)°
*V* = 1108.5 (5) Å^3^
*Z* = 4
Mo *K*α radiation
μ = 0.10 mm^−1^
*T* = 273 (2) K
0.30 × 0.26 × 0.25 mm

#### Data collection

Bruker SMART CCD area-detector diffractometer
Absorption correction: multi-scan (*SADABS*; Bruker, 2002 ▶) *T*_min_ = 0.972, *T*_max_ = 0.978
7124 measured reflections
1952 independent reflections
1624 reflections with *I* > 2σ(*I*)
*R*_int_ = 0.030

#### Refinement

*R*[*F* ^2^ > 2σ(*F* ^2^)] = 0.036
*wR*(*F* ^2^) = 0.102
*S* = 1.07
1952 reflections
153 parameters
H atoms treated by a mixture of independent and constrained refinement
Δρ_max_ = 0.19 e Å^−3^
Δρ_min_ = −0.14 e Å^−3^

### 

Data collection: *SMART* (Bruker, 2002 ▶); cell refinement: *SAINT* (Bruker, 2002 ▶); data reduction: *SAINT*; program(s) used to solve structure: *SHELXS97* (Sheldrick, 2008 ▶); program(s) used to refine structure: *SHELXL97* (Sheldrick, 2008 ▶); molecular graphics: *SHELXTL* (Sheldrick, 2008 ▶); software used to prepare material for publication: *SHELXTL*.

## Supplementary Material

Crystal structure: contains datablocks I, global. DOI: 10.1107/S1600536808024264/zl2130sup1.cif

Structure factors: contains datablocks I. DOI: 10.1107/S1600536808024264/zl2130Isup2.hkl

Additional supplementary materials:  crystallographic information; 3D view; checkCIF report

## Figures and Tables

**Table 1:** Hydrogen-bond geometry (Å, °)

*D*—H⋯*A*	*D*—H	H⋯*A*	*D*⋯*A*	*D*—H⋯*A*
N2—H2*A*⋯O2^i^	0.89 (2)	2.01 (2)	2.8864 (17)	169.0
C4—H4⋯*Cg*1^ii^	0.93	2.87	3.6488 (19)	142

Symmetry codes: (i) 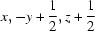; (ii) 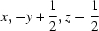. *Cg*1 is the centroid of the benzene ring.

**Fig. 1.**
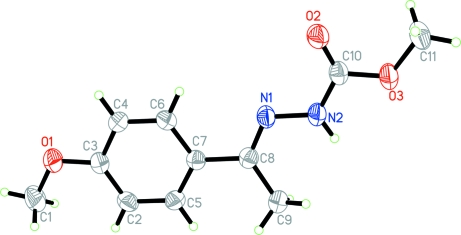
Molecular structure of (I), showing 50% probability displacement ellipsoids and the atomic numbering.

**Fig. 2.**
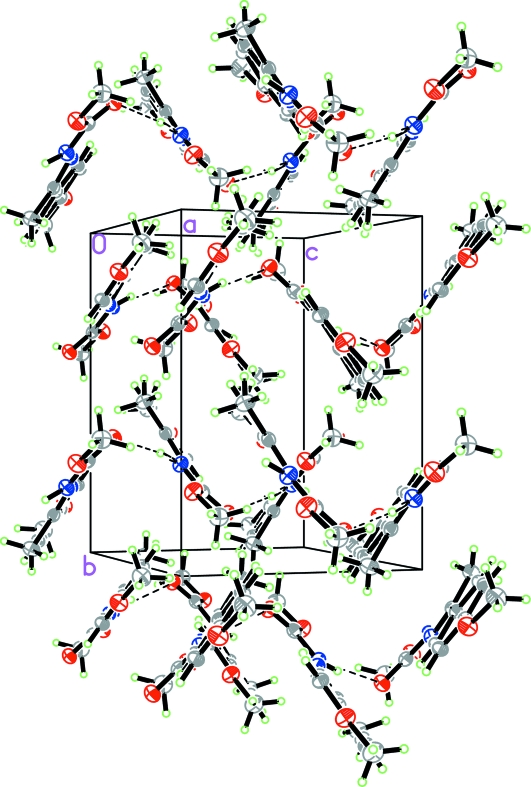
Crystal packing of the title compound, viewed approximately down the a axis. Dashed lines indicate intermolecular hydrogen bonds.

## supplementary crystallographic information

### Comment

Benzaldehydehydrazone derivatives have received considerable attention for a long time due to their pharmacological activity (Parashar *et al.*, 1988) and their photochromic properties (Hadjoudis *et al.*, 1987). Meanwhile, it's an important intermidiate of 1,3,4-oxadiazoles, which have been reported to be versatile compounds with many interesting properties (Borg *et al.*, 1999). As a further investigation of this type of derivatives, the crystal structure of the title compound, C_11_H_14_N_2_O_3_ (Fig.1), is described here.

The title molecule (Fig.1) adopts a *trans* configuration with respect to the C═N bond. The N1/N2/O2/O3/C10/C11 plane of the hydrazine carboxylic acid methyl ester group is slightly twisted away from the attached ring. The dihedral angle between the C2—C7 ring and the N1/N2/O2/O3/C10/C11 plane is 12.06 (9)°. The bond lengths and angles agree with those observed for methyl *N*'-[(*E*)-4-methoxybenzylidene] hydrazinecarboxylate (Shang *et al.*, 2007).

The molecules are linked into a one-dimensional network by N–H···O hydrogen bonds and C–H···π interactions (Table 1, Fig.2). The benzene rings of the inversion-related molecules are stacked with their centroids separated by a distance of 3.777 (1) Å, indicating π-π interactions.

### Experimental

4-Methoxy-acetophenone (1.50 g, 0.01 mol) and methyl hydrazinecarboxylate (0.90 g, 0.01 mol) were dissolved in stirred methanol (15 ml) and left for 3.5 h at room temperature. The resulting solid was filtered off and recrystallized from ethanol to give the title compound in 80% yield. Crystals suitable for X-ray analysis were obtained by slow evaporation of an ethanol solution at room temperature (m.p. 470–472 K).

### Refinement

The H atoms attached N2 were located in a difference map and its position and Uiso values were freely refined. C-bound H atoms were positioned geometrically (C—H = 0.93 or 0.96 Å) and refined using a riding model, with *U*_iso_(H) = 1.2 or 1.5*U*_eq_(C).

### Crystal data

**Table:**

C_11_H_14_N_2_O_3_	*F*_000_ = 472
*M**_r_* = 222.24	*D*_x_ = 1.332 Mg m^−^^3^
Monoclinic, *P*2_1_/*c*	Mo *K*α radiation λ = 0.71073 Å
Hall symbol: -P 2ybc	Cell parameters from 1952 reflections
*a* = 12.416 (3) Å	θ = 1.6–25.0º
*b* = 11.113 (3) Å	µ = 0.10 mm^−^^1^
*c* = 8.073 (2) Å	*T* = 273 (2) K
β = 95.628 (3)º	Block, colourless
*V* = 1108.5 (5) Å^3^	0.30 × 0.26 × 0.25 mm
*Z* = 4	

### Data collection

**Table:**

Bruker SMART CCD area-detector diffractometer	1952 independent reflections
Radiation source: fine-focus sealed tube	1624 reflections with *I* > 2σ(*I*)
Monochromator: graphite	*R*_int_ = 0.030
*T* = 273(2) K	θ_max_ = 25.0º
φ and ω scans	θ_min_ = 1.7º
Absorption correction: multi-scan(SADABS; Bruker, 2002)	*h* = −14→14
*T*_min_ = 0.972, *T*_max_ = 0.978	*k* = −13→11
7124 measured reflections	*l* = −9→9

### Refinement

**Table:**

Refinement on *F*^2^	Hydrogen site location: inferred from neighbouring sites
Least-squares matrix: full	H atoms treated by a mixture of independent and constrained refinement
*R*[*F*^2^ > 2σ(*F*^2^)] = 0.036	*w* = 1/[σ^2^(*F*_o_^2^) + (0.0461*P*)^2^ + 0.2826*P*] where *P* = (*F*_o_^2^ + 2*F*_c_^2^)/3
*wR*(*F*^2^) = 0.102	(Δ/σ)_max_ = 0.001
*S* = 1.07	Δρ_max_ = 0.19 e Å^−^^3^
1952 reflections	Δρ_min_ = −0.14 e Å^−^^3^
153 parameters	Extinction correction: SHELXL97 (Sheldrick, 2008), Fc^*^=kFc[1+0.001xFc^2^λ^3^/sin(2θ)]^-1/4^
Primary atom site location: structure-invariant direct methods	Extinction coefficient: 0.042 (4)
Secondary atom site location: difference Fourier map	

### Special details

**Table:**

Geometry. All esds (except the esd in the dihedral angle between two l.s. planes) are estimated using the full covariance matrix. The cell esds are taken into account individually in the estimation of esds in distances, angles and torsion angles; correlations between esds in cell parameters are only used when they are defined by crystal symmetry. An approximate (isotropic) treatment of cell esds is used for estimating esds involving l.s. planes.
Refinement. Refinement of F^2^ against ALL reflections. The weighted R-factor wR and goodness of fit S are based on F^2^, conventional R-factors R are based on F, with F set to zero for negative F^2^. The threshold expression of F^2^ > 2sigma(F^2^) is used only for calculating R-factors(gt) etc. and is not relevant to the choice of reflections for refinement. R-factors based on F^2^ are statistically about twice as large as those based on F, and R- factors based on ALL data will be even larger.

### Fractional atomic coordinates and isotropic or equivalent isotropic displacement parameters (Å^2^)

**Table:**

	*x*	*y*	*z*	*U*_iso_*/*U*_eq_	
H2A	0.6796 (14)	0.1822 (17)	0.308 (2)	0.059 (5)*	
C7	0.96977 (11)	0.12453 (12)	0.16378 (16)	0.0297 (3)	
C8	0.85779 (11)	0.12580 (12)	0.21369 (16)	0.0317 (3)	
C10	0.62251 (11)	0.29834 (13)	0.14528 (18)	0.0348 (3)	
C3	1.18244 (11)	0.12536 (13)	0.07457 (18)	0.0341 (3)	
C5	1.04408 (11)	0.03686 (13)	0.22113 (17)	0.0351 (4)	
H5	1.0223	−0.0236	0.2902	0.042*	
C6	1.00522 (11)	0.21282 (12)	0.05809 (17)	0.0348 (4)	
H6	0.9574	0.2727	0.0174	0.042*	
C2	1.14990 (11)	0.03671 (13)	0.17850 (18)	0.0375 (4)	
H2	1.1983	−0.0225	0.2196	0.045*	
C4	1.10911 (11)	0.21306 (13)	0.01308 (18)	0.0364 (4)	
H4	1.1305	0.2720	−0.0587	0.044*	
C1	1.36336 (12)	0.05169 (17)	0.0921 (2)	0.0551 (5)	
H1A	1.3691	0.0574	0.2113	0.083*	
H1B	1.4320	0.0704	0.0531	0.083*	
H1C	1.3425	−0.0286	0.0588	0.083*	
C11	0.44286 (12)	0.36579 (16)	0.1528 (2)	0.0528 (5)	
H11A	0.4628	0.4492	0.1589	0.079*	
H11B	0.3810	0.3529	0.2132	0.079*	
H11C	0.4254	0.3433	0.0385	0.079*	
O1	1.28412 (8)	0.13431 (10)	0.02268 (14)	0.0460 (3)	
O3	0.53202 (8)	0.29346 (10)	0.22464 (14)	0.0467 (3)	
O2	0.63408 (8)	0.36576 (10)	0.03058 (13)	0.0432 (3)	
N1	0.79718 (9)	0.21424 (11)	0.15959 (14)	0.0350 (3)	
N2	0.69424 (10)	0.21707 (12)	0.21353 (17)	0.0391 (3)	
C9	0.82244 (12)	0.02704 (14)	0.3229 (2)	0.0439 (4)	
H9A	0.8329	0.0521	0.4372	0.066*	
H9B	0.8646	−0.0439	0.3082	0.066*	
H9C	0.7472	0.0098	0.2933	0.066*	

### Atomic displacement parameters (Å^2^)

**Table:**

	*U*^11^	*U*^22^	*U*^33^	*U*^12^	*U*^13^	*U*^23^
C7	0.0318 (7)	0.0303 (7)	0.0271 (7)	−0.0005 (5)	0.0034 (5)	−0.0038 (5)
C8	0.0343 (7)	0.0334 (8)	0.0277 (7)	−0.0011 (6)	0.0052 (5)	−0.0037 (6)
C10	0.0317 (7)	0.0391 (8)	0.0346 (8)	0.0006 (6)	0.0082 (6)	−0.0059 (7)
C3	0.0301 (7)	0.0370 (8)	0.0357 (8)	0.0004 (6)	0.0048 (6)	−0.0050 (6)
C5	0.0388 (8)	0.0333 (8)	0.0336 (8)	0.0002 (6)	0.0058 (6)	0.0038 (6)
C6	0.0342 (7)	0.0328 (8)	0.0374 (8)	0.0051 (6)	0.0041 (6)	0.0021 (6)
C2	0.0362 (8)	0.0357 (8)	0.0403 (8)	0.0086 (6)	0.0021 (6)	0.0019 (6)
C4	0.0361 (8)	0.0354 (8)	0.0386 (8)	−0.0008 (6)	0.0079 (6)	0.0049 (6)
C1	0.0332 (8)	0.0649 (12)	0.0680 (12)	0.0125 (8)	0.0084 (8)	0.0035 (9)
C11	0.0314 (8)	0.0592 (11)	0.0684 (12)	0.0078 (7)	0.0075 (7)	0.0000 (9)
O1	0.0304 (5)	0.0522 (7)	0.0565 (7)	0.0053 (5)	0.0107 (5)	0.0060 (5)
O3	0.0316 (5)	0.0567 (7)	0.0539 (7)	0.0066 (5)	0.0153 (5)	0.0079 (5)
O2	0.0425 (6)	0.0493 (7)	0.0396 (6)	0.0093 (5)	0.0125 (5)	0.0054 (5)
N1	0.0308 (6)	0.0412 (7)	0.0345 (7)	0.0028 (5)	0.0097 (5)	0.0002 (5)
N2	0.0330 (6)	0.0467 (8)	0.0393 (7)	0.0047 (5)	0.0131 (5)	0.0059 (6)
C9	0.0379 (8)	0.0442 (9)	0.0516 (9)	0.0011 (7)	0.0144 (7)	0.0075 (7)

### Geometric parameters (Å, °)

**Table:**

C7—C5	1.3896 (19)	C2—H2	0.9300
C7—C6	1.399 (2)	C4—H4	0.9300
C7—C8	1.4850 (19)	C1—O1	1.4201 (19)
C8—N1	1.2878 (18)	C1—H1A	0.9600
C8—C9	1.500 (2)	C1—H1B	0.9600
C10—O2	1.2105 (17)	C1—H1C	0.9600
C10—N2	1.3476 (19)	C11—O3	1.4429 (19)
C10—O3	1.3479 (17)	C11—H11A	0.9600
C3—O1	1.3722 (17)	C11—H11B	0.9600
C3—C2	1.380 (2)	C11—H11C	0.9600
C3—C4	1.392 (2)	N1—N2	1.3905 (16)
C5—C2	1.390 (2)	N2—H2A	0.891 (19)
C5—H5	0.9300	C9—H9A	0.9600
C6—C4	1.3740 (19)	C9—H9B	0.9600
C6—H6	0.9300	C9—H9C	0.9600
			
C5—C7—C6	117.16 (13)	O1—C1—H1A	109.5
C5—C7—C8	121.66 (12)	O1—C1—H1B	109.5
C6—C7—C8	121.18 (12)	H1A—C1—H1B	109.5
N1—C8—C7	116.53 (12)	O1—C1—H1C	109.5
N1—C8—C9	124.24 (13)	H1A—C1—H1C	109.5
C7—C8—C9	119.23 (12)	H1B—C1—H1C	109.5
O2—C10—N2	127.18 (13)	O3—C11—H11A	109.5
O2—C10—O3	123.74 (13)	O3—C11—H11B	109.5
N2—C10—O3	109.08 (13)	H11A—C11—H11B	109.5
O1—C3—C2	124.78 (13)	O3—C11—H11C	109.5
O1—C3—C4	115.44 (13)	H11A—C11—H11C	109.5
C2—C3—C4	119.78 (13)	H11B—C11—H11C	109.5
C7—C5—C2	122.07 (13)	C3—O1—C1	117.08 (12)
C7—C5—H5	119.0	C10—O3—C11	115.40 (12)
C2—C5—H5	119.0	C8—N1—N2	115.82 (12)
C4—C6—C7	121.48 (13)	C10—N2—N1	118.55 (13)
C4—C6—H6	119.3	C10—N2—H2A	117.4 (12)
C7—C6—H6	119.3	N1—N2—H2A	122.0 (11)
C3—C2—C5	119.33 (13)	C8—C9—H9A	109.5
C3—C2—H2	120.3	C8—C9—H9B	109.5
C5—C2—H2	120.3	H9A—C9—H9B	109.5
C6—C4—C3	120.17 (13)	C8—C9—H9C	109.5
C6—C4—H4	119.9	H9A—C9—H9C	109.5
C3—C4—H4	119.9	H9B—C9—H9C	109.5

### Hydrogen-bond geometry (Å, °)

**Table:**

*D*—H···*A*	*D*—H	H···*A*	*D*···*A*	*D*—H···*A*
N2—H2A···O2^i^	0.89 (2)	2.01 (2)	2.8864 (17)	169.0
C4—H4···Cg1^ii^	0.93	2.87	3.6488 (19)	142

Symmetry codes: (i) *x*, −*y*+1/2, *z*+1/2; (ii) *x*, −*y*+1/2, *z*−1/2.

## References

[bb1] Borg, S., Vollinga, R. C., Labarre, M., Payza, K., Terenius, L. & Luthman, K. (1999). *J. Med. Chem.* **42**, 4331–4342. 10.1021/jm990197+ 10543877

[bb2] Bruker (2002). *SADABS*, *SMART* and *SAINT* Bruker AXS Inc., Madison, Wisconsin, USA.

[bb3] Hadjoudis, E., Vittorakis, M. & Moustakali-Mavridis, J. (1987). *Tetrahedron*, **43**, 1345–1360.

[bb4] Parashar, R. K., Sharma, R. C., Kumar, A. & Mohan, G. (1988). *Inorg. Chim. Acta*, **151**, 201–208.

[bb5] Shang, Z.-H., Zhang, H.-L. & Ding, Y. (2007). *Acta Cryst.* E**63**, o3394.

[bb6] Sheldrick, G. M. (2008). *Acta Cryst.* A**64**, 112–122. 10.1107/S0108767307043930 18156677

